# Association between peripapillary scleral deformation and choroidal microvascular circulation in glaucoma

**DOI:** 10.1038/s41598-019-54882-9

**Published:** 2019-12-06

**Authors:** Da Young Shin, Soo Ji Jeon, Eun Kyoung Kim, Kyoung In Jung, Hae Young Lopilly Park, Chan Kee Park

**Affiliations:** Department of Ophthalmology, Seoul St. Mary’s Hospital, College of Medicine, The Catholic University of Korea, Seoul, Republic of Korea

**Keywords:** Optic nerve diseases, Risk factors

## Abstract

Peripapillary vessel density, which is reduced in eyes with glaucoma, has been proposed as a diagnostic tool for the desease and peripapillary choroidal microvasculature dropout(MvD) is considered one of pathophysiological manifestation of glaucomatous damage. However, little is known about the underlying pathogenic mechanism of dropout. According to recent studies, MvD is associated with structural changes in ONH structures. Therefore, we investigated the association between peripapillary scleral deformation and MvD. Data from 62 open-angle glaucoma (OAG) eyes with MvD and 36 eyes without MvD were analyzed in this study. And eyes with MvD were classified into two groups based on location: a juxtapapillary group and a non-juxtapapillary group for further analysis. More eyes with MvD had focal scleral deformation than did those without MvD (64.5% versus 2.8%; P < 0.001). Peripapillary choroidal thickness and focal scleral deformation were significantly associated with MvD. And juxtapapillary group was more associated with focal scleral deformation and coincidental RNFL defects than non-juxtapapillary groups. Peripapillary choroidal MvD was associated with the presence of scleral deformation, especially with juxtapapillary MvD, which was related to corresponding RNFL defects.

## Introduction

Microvasculature of the optic nerve head (ONH) has long been recognized as a contributing factor in glaucoma^1,2^. Recent studies using optical coherence tomography angiography (OCT-A) have identified peripapillary vessel density around the ONH and localized microvasculature dropout (MvD) in the deep peripapillary choroidal layer round the ONH^3–6^. Peripapillary vessel density, which is reduced in eyes with glaucoma, has been proposed as a diagnostic tool for the disease^6,7^. Peripapillary deep-layer MvD, defined as microvasculature damage within the peripapillary choroid or sclera, is of clinical importance^1,8–10^. Ichiyama *et al*.^5^ found that MvD is associated with a thinner retinal nerve fiber layer (RNFL) and poorer visual field (VF) parameters, and Suh *et al*.^4^ found that eyes with MvD had reduced VF sensitivity. Park *et al*.^11^ found that the extent of MvD is associated with the degree of glaucomatous damage at diagnosis. MvD may therefore be a pathophysiological manifestation of glaucomatous damage^11^. However, little is known about the underlying pathogenic mechanism of dropout.

According to recent studies, MvD is associated with structural changes in ONH structures including focal laminar cribrosa (LC) defects, thinner choroidal thickness, and beta or gamma peripapillary atrophy (PPA)^4,12,13^. However, its pathogenesis and the role of sclera around ONH are poorly understood.

Peripapillary choroidal microvasculature is supplied by the short posterior ciliary arteries^14^. Short ciliary arteries pierce the sclera around the entrance of the optic nerve in the laminar level^15^. We hypothesized that deformation at the sclera where it connects to the lamina around the ONH may interfere with blood flow from adjacent short posterior ciliary arteries in the ONH.

In the present study, we used SS-OCT to evaluate the peripapillary choroid and sclera in eyes with or without MvD. For more detailed analysis, we divided eyes with MvD into two groups and we conducted subanalysis for two groups: juxtapapillary MvD (continuous to the scleral lip of the optic disc) and non-juxtapapillary MvD (discontinuous to the scleral lip of the optic disc). Because the radial peripapillary choriocapillaris adjacent to the disc constitute important blood vessels for the ONH, and previous studies have shown that the most vulnerable area for pressure is the junction of the optic disc and sclera with peripapillary scleral thinning^16,17^.

## Results

In total, 117 eyes underwent OCT-A and SS-OCT examination. Of the 117 eyes, 13(16.2%) were excluded from further analysis because of the poor quality of OCT-A /S-OCT images and multiple MvD (present in both nonjuxtapapillary and juxtapapilary). And six eyes(5.1%) were excluded because of interobserver disagreement in MvD location or scleral deformation. The remaining 98 eyes were further analyzed. Interobserver agreements in MvD and Scleral deformation were excellent(MvD, k = 0.839;95% confidence interval[CI], 0.770–0.908; P < 0.001, Scleral deformation, k = 0.933;95% confidence interval[CI], 0.887–0.979; P < 0.001).

Table 1 compares the clinical characteristics of eyes with and without MvD. The MvD group showed a lower spherical equivalent (P < 0.001), longer axial length (P < 0.001), higher PPA-to-disc ratio (P < 0.001), higher proportion of CTT (P < 0.001), and higher proportion of peripapillary scleral deformation (notching) (P < 0.001) than the non-MvD group.

**Table 1:** Comparison of the demographics and test results between patients according to the presence of the MvD.

Variables	Without MvD, 36 eyes, 36 patients	With MvD, 62 eyes 62 patients	P value
Age, y	54.39 ± 12.48	50.37 ± 11.20	0.104
Sex, male/female	10/26	28/34	0.089
CCT, μm	519.59 ± 42.36	513.52 ± 40.68	0.508
history of refractive surgery	6 (16.7%)	12 (19.4%)	0.740
history of diabetics, n (%)	1 (2.8%)	2 (3.2%)	0.276
history of hypertension, n (%)	1 (2.8%)	9 (14.5%)	0.064
IOP, mmHg	13.78 ± 3.26	13.79 ± 2.56	0.976
Spherical equivalent, D	−1.79 ± 2.42	−4.47 ± 2.79	**<0.001**
Axial length, mm	24.52 ± 0.33	25.88 ± 1.2	**<0.001**
PPA + disc/disc ratio (%)	139.78 ± 38.06	187.69 ± 47.03	**<0.001**
Choroidal thickness thinning, n(%)	5 (13.9%)	45 (72.6%)	**<0.001**
Focal scleral deformation (notching), n (%)	1 (2.8%)	40 (64.5%)	**<0.001**
DH, n (%)	5 (14.3%)	8 (12.9%)	0.848
VF MD, dB	−3.63 ± 4.67	−4.62 ± 4.67	0.307
VF PSD, dB	4.60 ± 0.97	5.48 ± 4.23	0.328

Chi – squared test and independent samples t-test were used.

CCT = central corneal thickness; IOP = intraocular pressure; D = diopter; PPA = peripapillary atrophy; DH = disc hemorrhage; VF = visual field; MD = mean deviation; PSD = pattern standard deviation.

Statistically significant values appear in boldface.

The factors associated with the presence of MvD were determined using logistic regression analysis (Table 2). Univariate analyses showed spherical equivalent (P < 0.001), axial length (P < 0.001), presence of peripapillary scleral notching (P < 0.001), CTT (P < 0.001), and ratio of PPA to disc area (P < 0.001) were associated with the presence of MvD. In multivariate analyses, only the presence of choroidal thickness thinning (P = 0.023) and focal peripapillary scleral deformation (P = 0.002) were significantly associated with MvD (Table 2).

**Table 2:** Factors associated with the presence of microvasculature dropout (MvD).

Variables	Univariant model		Multivariant model	
	Odds ratio, 95% CI	P value	Odds ratio, 95% CI	P value
Age, y	0.97, 0.94–1.01	0.106		
Sex, male/female	0.47, 0.19–1.13	0.092		
CCT, μm	1.00, 0.99–1.01	0.504		
history of refractive surgery	1.20, 0.41–3.53	0.741		
history of diabetics, n(%)	1.17, 0.10–13.34	0.901		
history of hypertension, n(%)	5.94, 0.72–49.00	0.098		
IOP, mmHg	0.98, 0.85–1.14	0.800		
Spherical equivalent, D	0.65, 0.53–0.80	**<0.001**		
Axial length, mm	1.96, 1.35–2.84	**<0.001**	1.09, 0.67–1.77	0.737
PPA + disc/disc area, per 1 µm larger (%)	1.04, 1.02–1.06	**<0.001**	1.03, 1.00–1.05	0.086
Choroidal thickness thinning, n(%)	16.41, 5.48–49.16	**<0.001**	6.01, 1.28–28.29	**0.023**
Focal scleral deformation (notching), n(%)	63.64, 8.15–496.66	**<0.001**	31.01, 3.44–279.38	**0.002**
DH, n (%)	0.85, 0.27–2.96	0.848		
VF MD, dB	0.95, 0.86–1.05	0.305		
VF PSD, dB	1.06, 0.95–1.17	0.320		

CCT = central corneal thickness; IOP = intraocular pressure; D = diopter; PPA = peripapillary atrophy; DH = disc hemorrhage; VF = visual field; MD = mean deviation; PSD = pattern standard deviation.

Statistically significant values appear in boldface.

**Figure 1:**
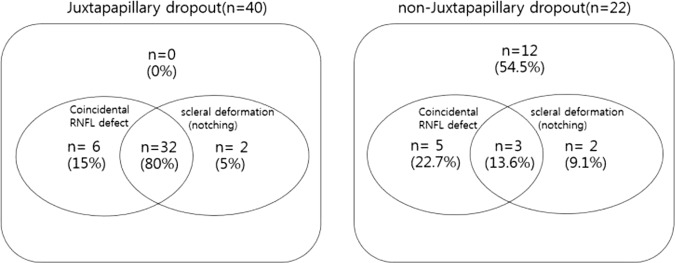
Venn diagrams showing the proportion of eyes that have coincidental RNFL defects and scleral deformation (notching) in juxtapapillary dropout and non-juxtapapillary dropout. The proportion of eyes that have both coincidental RNFL defects and scleral deformation (notching) was higher in the juxtapapillary group (n = 32, 80%) than in the non-juxtapapillary dropout (n = 3, 13.6%, p < 0.001, chi-squared test).

Table 3 compares the clinical characteristics of the juxtapapillary and non-juxtapapillary MvD groups. The difference in spherical equivalent and axial length between the two groups was not significant. The juxtapapillary MvD group showed a lower VF mean deviation, higher VF PSD, higher proportion of focal peripapillary scleral deformation (P < 0.001), higher proportion of matching of RNFL defects (P < 0.001), and presence of disc hemorrhage (P = 0.037). We also used logistic regression analysis to identify factors associated with the location of MvD in eyes. Univariate analyses showed scleral deformation (P < 0.001), VF MD (P = 0.012), VF PSD (P = 0.012), and matching of RNFL defects (P < 0.001) were significantly associated with juxtapapillary dropout (Table 4). Multivariate analyses revealed that only matching of RNFL defects and presence of scleral deformation were significantly associated with juxtapapillary dropout (Table 4). The Venn diagram (Fig. 1) details the distribution and overlap of coincidental RNFL defects and scleral deformation (notching) in juxtapapillary or non-juxtapapillary dropouts. The proportion of eyes that had coincidental RNFL defects and scleral deformation (notching) was higher in the juxtapapillary group (n = 32, 80%) than in the non-juxtapapillary group (n = 3, 13.6%, P < 0.001, X^2^).

**Table 3:** Comparison of the demographics and test results between patients according to the location of the MvD.

Variables	Non-juxtapapillary peripapillary MvD, 22 eyes	Juxtapapillary peripapillary MvD, 40 eyes	P value
Age, y	47.95 ± 10.27	49.14 ± 11.82	0.259
Sex, male/female	10/12	18/22	0.366
CCT, μm	521.00 ± 34.20	528.45 ± 26.49	0.437
Self-reported history of refractive surgery	6 (31.6%)	7 (20.0%)	0.342
Self-reported history of diabetics, n(%)	1 (4.5%)	1 (2.5%)	0.663
Self-reported history of hypertension, n (%)	2 (9.1%)	7 (17.5%)	0.368
IOP, mmHg	13.44 ± 1.79	14.30 ± 2.76	0.259
Spherical equivalent, D	−6.08 ± 1.97	−4.57 ± 2.76	0.057
Axial length, mm	26.18 ± 0.96	25.76 ± 1.32	0.257
PPA + disc/disc area, per 1 µm larger (%)	201.05 ± 30.82	181.76 ± 52.22	0.118
Choroidal thickness thinning, n(%)	17 (77.3%)	28 (70.0%)	0.539
Focal scleral deformation(notching), n (%)	5 (22.7%)	34 (86.1%)	**<0.001**
DH, n (%)	0 (0.0%)	7 (20.0%)	**0.037**
Spatial coincidental RNFL defect	8 (36.4%)	38 (95.0%)	**<0.001**
VF MD, dB	−2.59 ± 3.09	−5.79 ± 4.64	**0.007**
VF PSD, dB	3.44 ± 2.64	6.55 ± 4.50	**0.001**

Chi – squared test and independent samples t-test were used

CCT = central corneal thickness; IOP = intraocular pressure; D = diopter; PPA = peripapillary atrophy; DH = disc hemorrhage; RNFL = retinal nerve fiber layer; VF = visual field; MD = mean deviation; PSD = pattern standard deviation.

**Table 4:** Logistic regression testing factors associated with juxtapapillary MvD.

Variables	Univariant model		Multivariant model	
	Odds Ratio, 95% CI	P value	Odds ratio 95% CI	P value
age	1.03, 0.98–1.08	0.257		
sex	1.02, 0.36–2.90	0.973		
CCT, per 1	1.01, 0.99–1.03	0.428		
history of refractive surgery	0.57, 0.16–1.96	0.369		
history of diabetics, n(%)	0.54, 0.03–9.05	0.667		
history of hypertension, n(%)	2.12, 0.40–11.23	0.377		
IOP, per 1 mm Hg	1.16, 0.90–1.50	0.256		
Spherical equivalent, D	1.31, 0.98–1.76	0.068		
Axial length, mm	0.74, 0.44–1.25	0.256		
PPA + disc/disc, per 1 µm(%)	0.99, 0.98–1.00	0.132		
Choroidal thickness thinning, n(%)	0.69, 0.21–2.29	0.540		
Focal scleral deformation (notching), n(%)	19.27, 5.14–72.26	<0.001	21.25, 2.23–202.82	**0.008**
DH, n (%)	1.11*10^9^	0.999		
VF MD, dB	0.79, 0.65–0.95	0.012	0.87, 0.53–1.43	0.580
VF PSD, dB	1.25, 1.05–1.48	0.012	1.21, 0.84–1.75	0.305
Spatial coincidence of RNFL defect	33.25, 6.28–175.97	<0.001	39.29, 3.56–433.80	**0.003**

CCT = central corneal thickness; IOP = intraocular pressure; D = diopter; PPA = peripapillary atrophy; DH = disc hemorrhage; RNFL = retinal nerve fiber layer; VF = visual field; MD = mean deviation; PSD = pattern standard deviation.

Statistically significant values are shown in boldface.

## Discussion

MvD is a recently identified ocular condition that has been shown to indicate a localized perfusion defect in the peripapillary choroid^18^. MvD is usually found only within peripapillary atrophy on choroidal vessel density maps of the ONH generated by OCT-A^3,18^. PPA is seen mainly in myopic eyes, and PPA is usually larger in myopic eyes and increases in size with increasing myopia^19,20^. A number of studies suggest that increased mechanical stress on the ONH and peripapillary area during elongation of the globe may lead to compromised peripapillary deep-layer microvasculature in myopic eyes^21–23^. MvD appears to be closely related to myopia. However, multiple studies have reported that MvD is more closely related to glaucoma^24^ and MvD may be a pathophysiological manifestation of glaucomatous damage^3–5,25^. Shin *et al*.^24^ reported choroidal microvasculature dropout is not associated with myopia, but is associated with glaucoma. How this discrepancy arises? The exact causal mechanism of MvD and when it develops in glaucomatous eyes is unknown.

We used SS-OCT to characterize the sclera and choroidal thickness of the peripapillary deep-layer tissue in the area of the MvD, The results of multivariate analyses demonstrate that only scleral deformation (notching) and thinning of choroidal thickness were significant predictors of dropout. The juxtapapillary group was associated with a higher incidence of scleral deformation (notching) and greater spatial correlation of RNFL defects than the non-juxtapapillary group. This indicates that the presence of scleral deformation is related to MvD and glaucomatous optic neuropathy.

This raises the question of why scleral deformation arises and how it is related to MvD and glaucomatous damage such as RNFL defects. Numerous studies support a close connection between scleral biomechanics and ONH biomechanics and glaucoma^26^. For some time the conventional explanation was that, when intraocular pressure (IOP) increases, the LC deforms posteriorly, but the sclera remains undeformed^27,28^. However, more recent numerical studies suggest that the relationship between IOP and deformations of the LC and sclera is more complex^29–35^. Data from the majority of experimental modeling studies show that as IOP increases, the sclera deforms, and these deformations, when transmitted to the ONH, often have a significant influence on the displacement of the LC^33,34,36^. Through finite-element modeling of ONH biomechanics, Sigal^17^ reported that biomechanical effects in the LC depend strongly on scleral properties. In the thinner peripapillary atrophy region, IOP-induced deformation could have a larger impact on LC deformation. We hypothesized that extreme scleral deformation such as notching may account for the generation of dropout-causing closures of choriocapillaries passing through the sclera supply to the ONH. Because the laminar zone is supplied by branches from the short posterior ciliary artery, a closed short posterior ciliary artery due to scleral deformation reduces blood supply to the ONH and can accelerate glaucomatous damage. In summary, development of dropout may result from glaucomatous optic nerve damage, but it also can be a causative factor in the progression of glaucoma, and may account for the relationship between dropout and glaucoma.

**Figure 2:**
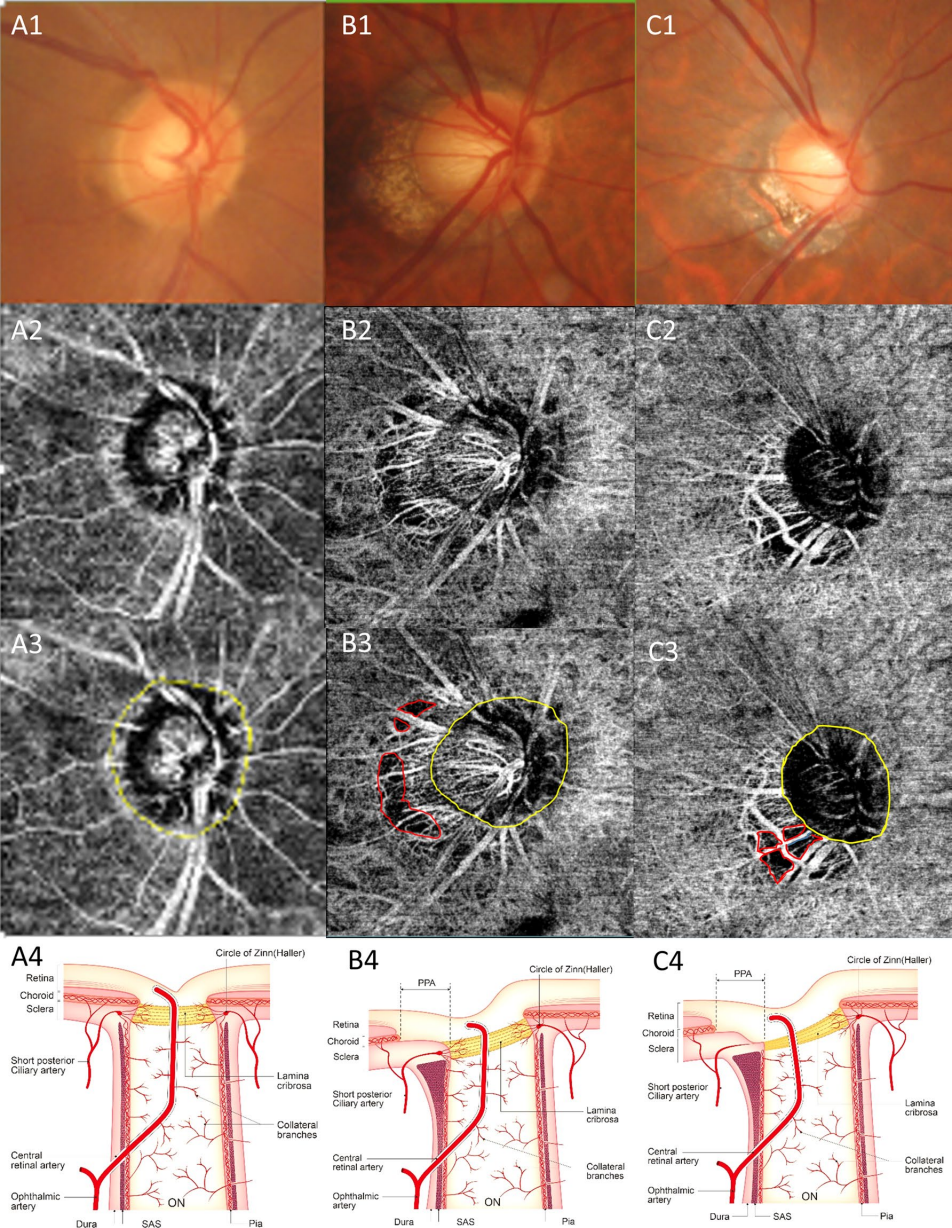
Determining the location of the MvD. (**A**) normal eye. There is no MvD. (**B**) non-juxtapapillary MvD in the right eye. Well-demarcated deep-layer MvD (**B3**, red dotted line). The yellow line is the margin of the scleral lip in the optic disc. The red line is not connected to the yellow line (**B3**). C. juxtapapillary MvD in the right eye. Well-demarcated deep-layer microvasculature dropout (red dotted line). The yellow line is the margin of the scleral lip in the optic disc. The red line is connected to the yellow line **C3**). The schematic layout of normal eye, non-juxtapapillary and juxtapapillary MvD (**A4,B4,C4**). The vessel and choroid tissue are stretched and scanty due to scleral stretching (**B4**). There is closure of the choriocapillaris due to scleral deformation around optic disc (**C4**).

**Figure 3:**
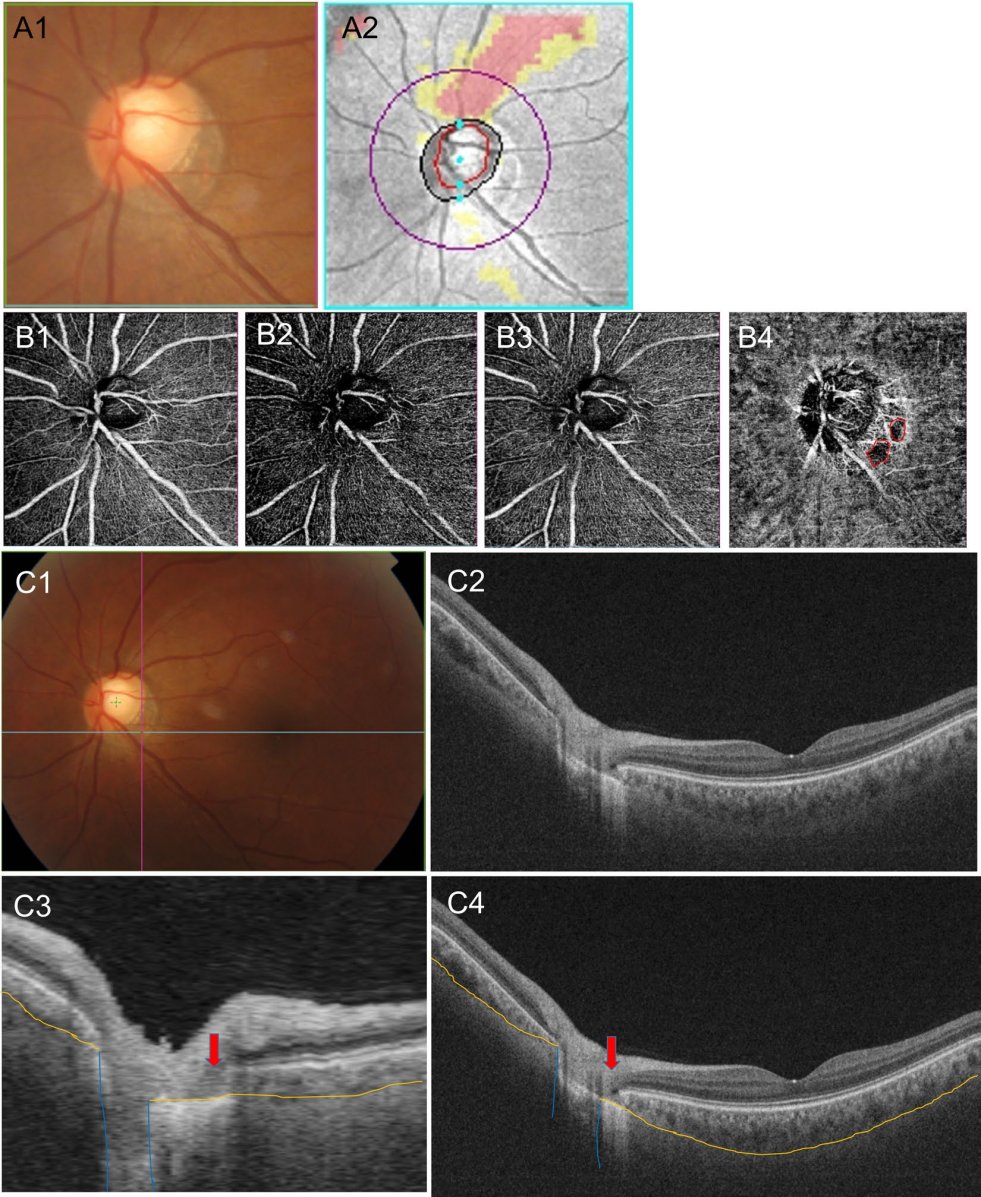
A representative case. A 44-year-old man with open-angle glaucoma had non-juxtapapillary dropout. (**A**) A localized, superotemporal retinal nerve fiber layer defect in the left eye (12 o’clock) (**B**). OCT angiography, the superficial layer (**B1**), vitreoretinal layer (**B2**), radial capillary network (**B3**), and choroidal microvasculature (**B4**). A microvasculature dropout is observed in the non-juxtapapillary area. Well-demarcated deep-layer microvasculature dropout (red dotted line, 4~5 o’clock) (**B4**). The location of dropout (4~5 o’clock) does not match the location of the RNFL defect (12 o’clock). (**C**) Green solid lines indicate locations of the horizontal B-scan (**C1**). Purple solid lines indicate locations of the longitudinal B-scan (**C1**). Horizontal B-scan (**C2,C4**). Longitudinal B-scan (**C3**). The red arrow indicates the location of microvasculature dropout (**C3,C4**). The orange line indicates the chorioscleral interface and the blue line indicates the optic canal (**C3,C4**). There is no deformity or defect-like depressed lesion (groove, notch) in the yellow line (**C3,C4**).

**Figure 4:**
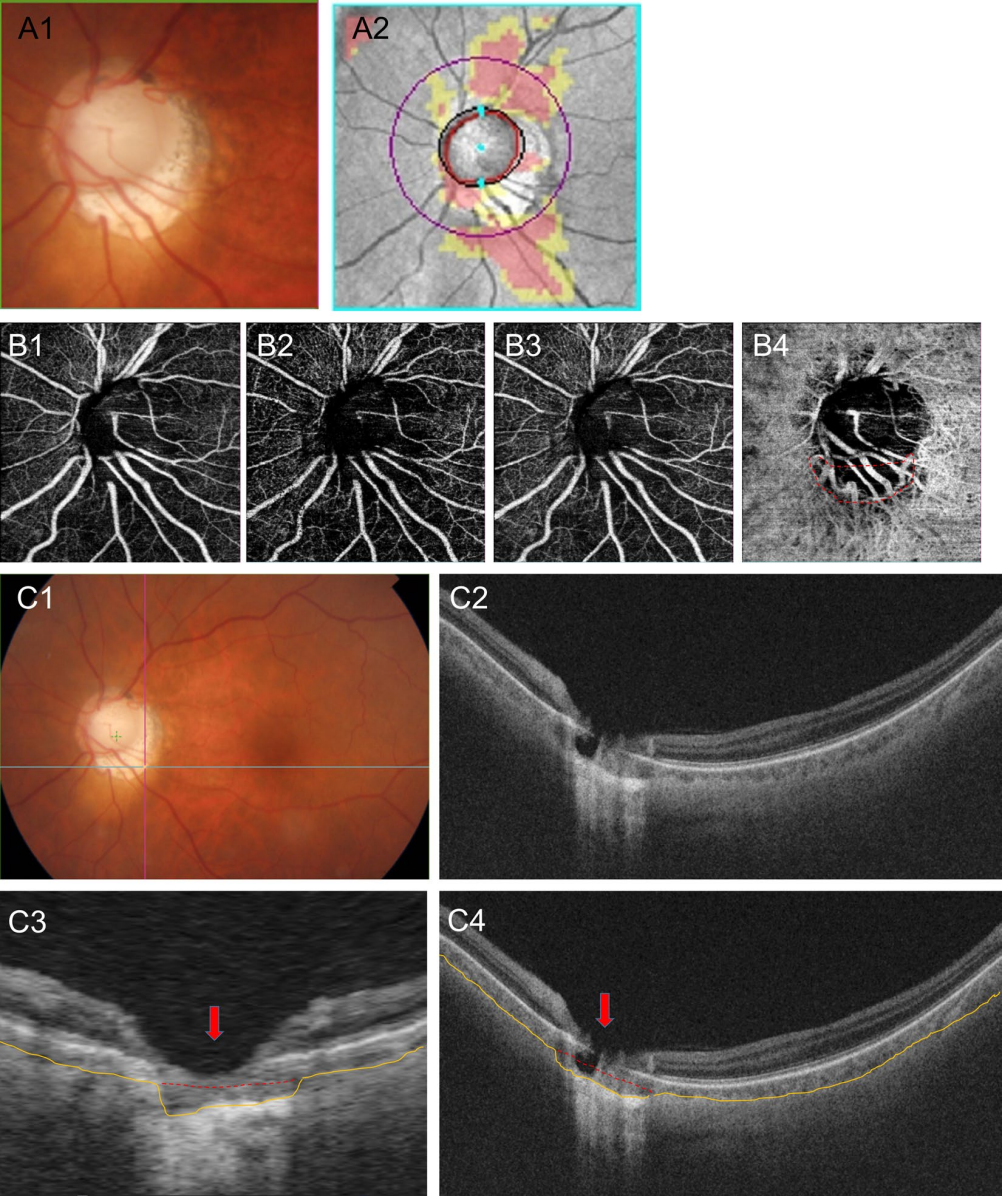
A representative case. A 64-year-old man with open-angle glaucoma had juxtapapillary dropout. (**A**) A localized, inferotemporal retinal nerve fiber layer defect in the left eye (4~7 o’clock). (**B**) OCT angiography, the superficial layer (**B1**), vitreoretinal layer (**B2**), radial capillary network (**B3**), and choroidal microvasculature (**B4**). A microvasculature dropout is observed in the juxtapapillary area. Well-demarcated deep-layer microvasculature dropout (red dotted line, 4~7 o’clock) (**B4**). The location of dropout (4~7 o’clock) match with location of the RNFL defect (4~7 o’clock). (**C**) Green solid lines indicate locations of the horizontal B-scan (**C1**). Purple solid lines indicate locations of the longitudinal B-scan (**C1**). Horizontal B-scan (**C2,C4**). Longitudinal B-scan (**C3**). The red arrow indicates the location of microvasculature dropout (**C3,C4**). The orange line indicates the chorioscleral interface line (anterior surface of the sclera) and the red dot line implies a non-depressed scleral line (**C3,C4**). The interface between the red and yellow lines implies a depressed space (**C3,C4**).

The relationship between PPA and dropout has been the subject of several studies. Sung *et al*.^13^ found beta-zone PPA width was closely correlated with deep peripapillary vessel density. Suh *et al*.^12^ found peripapillary deep-layer microvasculature dropout was associated with the presence and larger width of rPPA, while the presence and width of the exposed scleral flange may be associated with deep-layer MvD. Which PPA features are significant remains unclear. We conducted subanalysis for two groups: a juxtapapillary group (dropout connected to the scleral lip) and a non-juxtapapillary group (dropout unconnected to the scleral lip).Because we think more important blood vessels for ONH are radial peripapillary choriocapillaris adjacent to the disc and more vulnerable area for pressure was junction of optic disc and sclera. In the present study, juxtapapillary dropout was associated more closely with focal scleral deformation than was non-juxtapapillary dropout. And only the juxtapapillary dropout group showed RNFL defects in the dropout location. We hypothesized that in the case of juxtapapillary dropout, MvD is caused by closure of the choriocapillaris due to focal extreme scleral deformation (Fig. 2C-3). However, in the non-juxtapapillary dropout group, MvD was caused by stretching of the vessel and thinning of choroid tissue due to scleral stretching during elongation of the globe (Fig. 2B-3). The schematic layout is shown in Fig. 2 and representative cases are shown in Figs. 3 and 4. This means even stretching of the peripapillary sclera during myopic eyeball elongation may cause choroidal microcirculation disturbance which has little effect on RNFL damage. However, when focal changes of the peripapillary sclera occur in myopic eyes, this could be related to juxtapapillary MvD and RNFL damage could be affected. This difference was independent of the degree of myopia. Therefore, focal changes to the peripapillary sclera are important subjects of investigation when studying the effects of myopia on RNFL damage in glaucoma.

In the first representative case, a 44-year-old man with open-angle glaucoma had non-juxtapapillary MvD (Fig. 3). A localized, superotemporal retinal nerve fiber layer defect was detected at 12 o’clock hour in his left eye (Fig. 3A-2). But the location of MvD (4~5 o’clock hour) did not match the location of the RNFL defect (Figs. 3A-2 and 3B-4), and the anterior surface of the sclera was smoothly connected to the optic canal. However, in another case, a 64-year-old man with open-angle glaucoma had juxtapapillary dropout (Fig. 4). A localized, inferotemporal retinal nerve fiber layer defect was detected at 4~7 o’clock hour in his left eye (Fig. 4A-2) and microvasculature dropout was observed in the juxtapapillary area. The location of dropout (red dotted line, 4~7 o’clock hour) matched the location of the RNFL defect (Fig. 4A-2, B-4). The yellow line indicates the chorioscleral interface line (anterior surface of the sclera) and the red line implies a non-depressed scleral line (Figs. 4C-3,4). Between the red line and the yellow line is the area where the sclera likely depressed and flattened.

The numerous studies showed that eyes with MvD had significantly worse VF sensitivity and thinner RNFL thickness than those without MvD^3,6,37^. In our study, the eyes with or without dropout did not show significant differences in MD and PSD of VF(Tables 1 and 2). We think that glaucomatous damage will progress further in eyes with MvD than without MvD. However, this does not mean that all eyes with MvD have worse VF or thinner RNFL thickness than all eyes without MvD. Therefore, the clear difference can be seen when comparing before and after MvD occurences in the same eye. In other study, there is no statistical difference in baseline MD values of VF and baseline RNFL thickness between the groups with and without MvD, although there was a difference in the rate of RNFL thinning (mm/yr)^11^. Since a good MD value at one period does not predict the low risk of future glaucomatous damage, longitudinal study might be necessary.

In conclusion, patients with scleral deformation around the optic nerve head are associated with MvD, and juxtapapillary MvD in particular, a condition related to a glaucomatous optic neuropathy-like RNFL defect.

## Materials and Methods

### Study subjects

This study included 98 cases of open-angle glaucoma (OAG) who visited the Seoul St. Mary’s Hospital between July and October 2018. The work was approved by the Institutional Review Board of the Catholic University of Korea, Seoul, Korea and we followed all relevant tenets of the Declaration of Helsinki. Informed consent was obtained from all the subjects.

OAG was defined as the presence an open-angle sign of glaucomatous optic nerve damage, such as diffuse or localized rim thinning, disc hemorrhage, a notch in the rim, a vertical cup-to-disc ratio higher than that of the other eye by more than 0.2, or compatible repeated VF damage. Glaucomatous VF damage was defined as a VF outside the normal limits on the glaucoma hemifield test with a pattern standard deviation (PSD) outside 95% of normal limits as confirmed by two consecutive, reliable tests (≤33% fixation losses and <15% false positives).

All enrolled patients underwent a complete ophthalmic examination, including measurement of best-corrected visual acuity, intraocular pressure, refraction, slit-lamp biomicroscopy, gonioscopy, central corneal thickness via ultrasound pachymetry (Tomey Corp, Nagoya, Japan), axial length via ocular biometry (IOLMaster, Carl Zeiss Meditec, Dublin, CA), as well as a dilated stereoscopic examination of the optic disc, red-free fundus photography (Kowa nonmyd WX; Kowa Company Ltd., Tokyo,Japan), Cirrus OCT (Carl Zeiss Meditec), Humphrey VF examination using the Swedish interactive threshold standard 24-2 algorithm (Carl Zeiss Meditec), a review of medical history, swept-source OCT, and OCT-A (DRI OCT Triton; Topcon corporation Tokyo, Japan) examinations. The RNFL defect locations were determined as clock hours. Data were reviewed by two of the authors (D.Y.S and H.Y.P).

### OCT angiography

The ONH and peripapillary region were imaged using a commercial, swept-source OCT-A device. Scans were taken from 4.5 × 4.5 mm cubes. The peripapillary microvasculature in deep layers was based on automated layer segmentation performed by the built-in OCT software. The en-face images of the deep layer were derived from an en-face slab, extending from the retinal pigment epithelium to 390 µm below Bruch’s membrane. The technique can show full thickness of the choroid and the inner scleral surface. MvD was defined as a focal complete loss of the choriocapillary or the microvasculature on both horizontal and vertical and radial within the visible microvascular network. To avoid false positives, MvD was identified when the dropout width was greater than twice that of the visible juxtapapillary microvessels on the peripapillary choroidal microvasculature map^11^. To avoid false negatives, reflectance or shadowing of the large vessels was excluded from the qualitative review^4^. Juxtapapillary dropout occurred when the location of dropout was connected to the scleral lip of the optic disc (Fig. 2C). If the dropout was apart from the scleral lip of the optic disc, it was considered a non-juxtapapillary dropout (Fig. 2B). To clarify the difference the two, the non-juxtapapillary dropout included at least 500 micrometer distances from the scleral lip. Two independent observers blinded to clinical data (D.Y.S and H.Y.P), independently identified all MvDs. The subject was excluded from the analysis if consensus between the two observers could not be reached

### Swept-source optical coherence tomography

SS-OCT was performed at a scan speed of 100,000 A-scan/s. The center wavelength of the beam was 1050 nm to ensure penetration through cataracts and hemorrhages. Because of the high penetration of 1-µm light, *in vivo* imaging of the human sclera was possible with SS-OCT^37,38^. Repeated experiments confirmed that 1-μm SS-OCT reached the sclera^38,39^. SS-OCT also revealed the line of chorioscleral interface (anterior surface of sclera), produced a pseudocolor map of choroidal thickness of the *in vivo* human macula and ONH, and provided an intuitive and comprehensive understanding. Detailed specifications of the SS-OCT have previously been described^37,38^. Thinning of choroidal thickness (CTT) occurs when the thickness of the peripapillary choroid is below 50 micrometers within the area of the choroidal lumen and 500 μm from the border tissue of Elschnig^40^. That value was defined based on previous study on choroidal thickness, which showed that the mean choroidal thickness in normal eye was 135.56 (±42.66)^40^. The value (50 micrometers) is less than 2 standard deviation from mean^40^.

Peripapillary scleral deformation (notching) is defined as small grooves or an area focally more depressed than elsewhere. To be classified as a scleral deformation, the size of notching was required to be >100 µm in depth and >50 µm in diameter according to at least two consecutive scans. These criteria were used to reduce the possibility that the scleral deformations were due to vascular shadowing on the en-face SS-OCT images. Figure 3 is an image of an eye without peripapillary scleral deformation and Fig. 4 is an image of eyes with peripapillary scleral deformation.

### Statistical analysis

Descriptive statistics were calculated as the mean and standard deviation and a chi-squared test was used to compare categorical variables. The extent of interobserver agreement in terms of MvD determination and scleral deformation were assessed using kappa statistics (k value). Univariate and multivariate logistic regression analysis were used to identify factors associated with the presence of MvD. A value of P < 0.05 indicated statistical significance. All statistical analyses were performed using SPSS for Windows (v. 24.0; SPSS Inc, Chicago, Illinois, USA). Subanalysis was done between the two groups (non-juxtapapillary dropout and juxtapapillary dropout).
